# The Online Ordering Behaviors among Participants in the Oklahoma Women, Infants, and Children Program: A Cross-Sectional Analysis

**DOI:** 10.3390/ijerph19031805

**Published:** 2022-02-05

**Authors:** Qi Zhang, Kayoung Park, Junzhou Zhang, Chuanyi Tang

**Affiliations:** 1 School of Community and Environmental Health, Old Dominion University, Norfolk, VA 23529, USA; 2 Department of Mathematics and Statistics, Old Dominion University, Norfolk, VA 23529, USA; kypark@odu.edu; 3 Department of Marketing, Montclair State University, Montclair, NJ 07043, USA; zhangju@montclair.edu; 4 Department of Marketing, Old Dominion University, Norfolk, VA 23529, USA; ctang@odu.edu

**Keywords:** benefit redemption, COVID-19, grocery store, online ordering, WIC

## Abstract

The Special Supplemental Nutrition Program for Women, Infants, and Children (WIC) is a nutrition assistance program in the United States (U.S.). Participants in the program redeem their prescribed food benefits in WIC-authorized grocery stores. Online ordering is an innovative method being pilot-tested in some stores to facilitate WIC participants’ food benefit redemption, which has become especially important in the COVID-19 pandemic. The present research aimed to examine the online ordering (OO) behaviors among 726 WIC households who adopted WIC OO in a grocery chain, XYZ (anonymous) store, in Oklahoma (OK). These households represented approximately 5% of WIC households who redeemed WIC benefits in XYZ stores during the study period, which was 1 July to 31 December 2020. This period was during the COVID-19 pandemic but after the temporary lockdown in Oklahoma had been lifted. Descriptive statistics were estimated for WIC OO households’ adoption behaviors and their orders. The Cox proportional hazard model and zero-truncated negative binomial regression were applied to examine the relationship between participants’ socio-demographics and the length of time between 1 July 2020, and their first OO, as well as the number of WIC online orders. About 80% of these online orders were picked up without any changes. Minority households had a significantly longer time before adopting their first OO (hazard ratio (HR) < 1, *p* < 0.001), while households with a child or a woman participant, or more participants, had a shorter time before adopting OO (HR > 1, *p* < 0.05). Non-Hispanic black households had a fewer number of OOs than non-Hispanic white households (B = −0.374, *p* = 0.007). OO adoption varied across socio-demographics. More efforts are needed to ensure equal access and adoption of WIC OO.

## 1. Introduction

Malnutrition is a global threat to maternal and child health, but among developed countries, the United States (U.S.) is the only one with a widespread child hunger problem [1,2]. To provide nutrition assistance to low-income women, infants, and children under age 5, the United States Department of Agriculture (USDA) established the Special Supplemental Nutrition Program for Women, Infants, and Children (WIC) in 1974. In Fiscal Year 2019, the program covered approximately 6.4 million income-eligible pregnant, breastfeeding, and postpartum women as well as infants and children under age 5 [3]. Since the U.S. is unique among developed countries in having a large-scale malnutrition problem in the targeted population, there is no program comparable to WIC in other high-income countries.

WIC participants receive a list of food benefits from the WIC agencies and can redeem these benefits at WIC-authorized vendors [3]. To ensure the integrity of the redemption, WIC regulations require that food benefit transactions have to be completed in the presence of a cashier [4]. Consequently, this restriction prevents participants from redeeming their food benefits online using their WIC electronic benefit transfer (EBT) cards, although online redemption is acceptable in another federal nutrition assistance program, the Supplemental Nutrition Assistance Program (SNAP) [5]. This restriction has created more challenges for WIC participants during the coronavirus disease 2019 (COVID-19) pandemic when more U.S. consumers adopted online ordering (OO) for grocery shopping to reduce their infection risk [6,7].

To address this important issue, the USDA established a WIC task force to explore alternative models for redeeming WIC benefits [8]. At the same time, the American Rescue Plan Act of 2021 allocated USD 390 million to support WIC innovation and outreach [9]. Consequently, since WIC is a federally funded but state-operated program, a few WIC state agencies have pilot tested some innovative WIC OO models in the last two years. The Oklahoma (OK) WIC agency pilot tested an “online-ordering-store-pick-up” model in the XYZ (anonymous) grocery chain, where all food stocks are WIC-eligible. It is the only grocery chain in OK offering WIC online ordering. Any shoppers can use the XYZ app to order foods. WIC customers, however, cannot use the app to pay online but must pay for the food with their WIC EBT cards when picking up the order in the store or at the curbside.

Few studies have been published to evaluate these innovations. Two pioneering studies conducted qualitative research regarding OO experiments in Tennessee prior to the pandemic [10,11]. However, there are still no large-scale quantitative evaluations about WIC online ordering adoption behaviors. Therefore, it is timely and policy-significant to evaluate any preliminary efforts that have been made related to WIC OO.

Examining customer behaviors in XYZ’s WIC online ordering (XYZ-WIC-OO) in OK can provide two lines of preliminary but important evidence to WIC stakeholders, including WIC-authorized vendors, policymakers, and state agencies that may wish to design and implement WIC OO. First, WIC vendors care about OO customers’ redemption behaviors: since WIC OOs are not binding with any payment methods, e.g., credit card or WIC EBT card, changes in the orders, such as “no-shows” (i.e., shoppers order the food but do not pick it up) and last-minute changes in ordered items in-store or at curbside, can be disruptive to stores’ business operations. Second, WIC agencies need to understand the factors associated with WIC OO adoption and use so they can promote it among WIC households less inclined to adopt. For example, the proportion of one-time WIC OO users among all WIC customers might indicate whether the WIC OO process will be sustainable in the future. The time it takes a household to place their first OO can indicate the dissemination pace among WIC households, while the number of repeated online orders may imply WIC customers’ continued engagement with this new WIC redemption method [12]. This study aims to examine the patterns of WIC OO among OK WIC customers in XYZ stores. The hypothesis is that socio-demographics and WIC participation status can significantly affect the adoption of WIC OO.

## 2. Materials and Methods

### 2.1. Setting

WIC participants receive a list of food benefits, such as milk, cereal, or other nutritious food, prescribed by the WIC agencies according to federally specified nutritional standards, and they can redeem these food benefits in WIC-authorized stores within a fixed amount of time, usually a month [3]. Among WIC-authorized grocery stores, a special type of grocery store, the “WIC-only store,” stocks only WIC-eligible foods, mainly serves WIC customers, and receives the majority of its revenues from WIC redemptions [13]. XYZ is such a chain, with 10 stores in OK. XYZ stores serve approximately one-third of the WIC households in the state and receive one-fourth of the state’s total WIC food expenditures. Since July 2020, XYZ customers have been able to order foods on the XYZ app without making an online payment. Before placing an order, customers are required to provide their names and cell phone numbers and to choose an XYZ store and time to pick up their order. Customers can use their WIC EBT cards or any other payment methods when they pick up their orders in the store or at the curbside.

### 2.2. Data

The online ordering data from the XYZ app were linked with Oklahoma WIC administrative data at the household level (using the WIC household ID number) and, since the WIC OO was implemented during the pandemic, the data were also linked with the COVID-19 data by each WIC household’s residence county [14]. The online ordering data include information on each individual transaction, such as product identification number, description of the ordered item, number of units redeemed, and unit price. The WIC administrative data contain the participating households’ socio-demographic information, WIC participation statuses, WIC food benefit prescriptions, and redemption records (store, date, food items, and their redemption values). It should be noted that the redemption data does not provide direct information about the nutritional value of the food ordered or picked up. The COVID-19 data had the weekly incidence numbers of COVID-19 cases by counties in OK. Because online ordering was fully implemented in XYZ stores in July 2020, the study period was from 1 July 2020 to 31 December 2020.

WIC households who shop at XYZ stores represent approximately one-third of all WIC households in OK. This study included all WIC households who adopted at least one WIC OO in XYZ stores in the study period. Since XYZ stores are the only WIC-authorized stores in OK that allow WIC OO, our sample represents all WIC households who have used OO in OK so far. However, our sample could not represent any WIC households at the national level, because the WIC OO is still being piloted tested in a few states. Since the WIC program is operated at the state level, this type of store-based evaluation study targeting the WIC population has often been conducted at the store or local level [10,11].

### 2.3. Measures

#### 2.3.1. Online Ordering Behaviors

Since some households used online ordering only once in the study period, the number and the proportion of “one-timers” vs. repeated customers were calculated to indicate the general adoption behaviors among WIC households. Since all customers could order food benefits online sometimes but redeem benefits in-store at other times, just like exclusively non-OO customers, it was important to calculate the number of OOs and the proportion of OOs in all redemption efforts by each OO household.

Since payment was not made or temporarily held when placing an order, the OO offered by XYZ tended to have “no shows,” that is, customers who did not pick up their orders. Moreover, sometimes the final processed orders were different from the initial orders, e.g., some items were removed from an order, or some new items were added after the order was placed. Therefore, four final order process statuses were created: exact order picked up, orders picked up with added items but no missing items, orders picked up with missing items but no added items, and orders picked up with both missing and added items. These four statuses indicated different levels of complexity for store staff in trying to fulfill the orders. Moreover, the time (number of days) from initiation of the app on 1 July 2020, to the first online ordering date was calculated for each household to indicate the time needed for each household to try OO for the first time.

#### 2.3.2. Confounding Factors

The socio-demographics of WIC households included race/ethnicity, which was determined by the eldest WIC participant in the household; whether there was an infant, child, or woman participant in the household; the number of WIC participants; the household size; and the existing customer status, i.e., whether the household had redeemed their WIC benefits at XYZ before 1 July 2020. Since the study took place during the pandemic, the analyses controlled the four-week moving-average COVID-19 incidence in the households’ residential counties and the county population as estimated in 2019 [15].

### 2.4. Data Analyses

Since this is secondary data analysis, no recruitment was conducted and no questionnaire was implemented. The analyses were intended to answer three main research questions: first, what was the socio-demographic composition of the WIC OO consumers; second, was there a significant difference in the WIC OO adoption behaviors and outcomes across socio-demographic groups; and finally, were the socio-demographic factors related to the WIC OO adoption time? The detailed steps of the data analyses were as follows:

First, we estimated the socio-demographics of WIC OO households in total and by their OO frequencies (i.e., repeat customers and one-timers). Second, the descriptive statistics of OO adoption outcomes, including time (number of days) until the first OO, the number of OOs per benefit cycle, and the proportion of OOs in all redemptions were also calculated by socio-demographics. Third, we calculated the average rate of the four final order statuses by socio-demographic groups. In these descriptive analyses, a Chi-square test and a Wilcoxon rank-sum test were applied to check for significant differences across socio-demographics.

Fourth, to analyze the factors contributing to the time until the first OO was placed, we applied survival analysis, which can handle censoring data and utilize time variables [16]. The Cox proportional hazards regression model was applied to conduct the survival analysis and to examine the relationship between socio-demographics and the time till the first OO. Notably, the conventional linear regression models would provide inconsistent estimates by ignoring or improperly using censored information due to the cutoff time period [16].

Finally, to understand how the number of OOs placed was related to socio-demographics, a multivariate regression model was applied. Since the outcome, number of OOs placed, is a count variable without zero values, we applied a zero-truncated negative binomial regression model [17]. R was used for analyses, and the significance level was set at *p* < 0.05 [18]. The study was approved by the Institutional Review Board at Old Dominion University.

## 3. Results

During July–December 2020, the OO orders were not differentiated between WIC or non-WIC customers, since there was no indicator in the ordering system for WIC participation status. The ordinary least squares (OLS) regression indicated that there was no significant relationship between the no-show rates and the weekly time trend.

Table 1 presents the socio-demographics of WIC online orders households. Overall, the racial/ethnic composition between one-timers and repeat consumers did not show significant differences. Moreover, there were no statistical differences between one-timers and repeat consumers for households with or without an infant, child, or woman participant. The composition of one-time and repeat consumers showed significant differences across households with different numbers of WIC participants, but the differences across household sizes were not significant.

Table 1 also presents the average number of days until the first OO and the average number of OOs across socio-demographic groups. Most of the socio-demographics had no significant relationship with the time to the first OO. It took significantly less time for the existing XYZ customers to place their first OOs than for new XYZ customers. Households with a child participant tended to place more OOs than households without a child participant. As the number of WIC participants in the household increased, the number of OOs also tended to increase. In terms of the rate of OO out of all redemption trips, the racial/ethnic groups showed significant differences. Additionally, the rate of OO for households with a child participant tended to be significantly lower than that for households without a child participant. Existing XYZ customers had a significantly lower rate of OO than new consumers.

Table 2 presents OO characteristics across socio-demographics. No significant disparities were detected across socio-demographics regarding the rate of exact orders. Racial/ethnic disparities existed regarding the rate of orders with added items but no missing items. In addition, households with an infant or woman participant tended to have a significantly higher rate of orders with added items but no missing items than households without an infant or woman participant, while households with or without a child participant reflected an opposite pattern. Furthermore, the rate of OOs with added items but no missing items was significantly related to the number of WIC participants in the household. In terms of the rate of OOs with missing items but no added items, only households with or without a child participant and whether the household was an existing XYZ customer or not showed a significant difference. Specifically, households with a child participant tended to have a significantly higher rate of OOs with missing items but no added items than households without a child participant. The existing consumers also had significantly higher rates than new consumers. No significant difference across socio-demographics was detected regarding the rate of orders with both missing items and new items added.

Table 3 presents the results of the Cox proportional hazards regression model for the time until the first use of WIC OO and the zero-truncated negative binomial regression model for the number of OOs. The COVID-19 incidence significantly impacted the time until the first use of WIC OO. As the number of COVID-19 cases increased in the county, time to the first OO seemed to increase. When compared with non-Hispanic whites, all minority groups had a hazard ratio significantly smaller than 1. This indicated that it took longer for non-Hispanic blacks, Hispanics, and other racial/ethnic groups of WIC customers to adopt WIC OO than for non-Hispanic whites. It took the households with a child or woman participant less time to place their first OOs than for those without a child or woman participant. Households with or without an infant participant did not show a statistical difference in time to the first OO. In addition, as the number of WIC participants in a household increased, the time to place the first OO seemed to decrease. However, as the household size increased, the time to place the first OO tended to increase. The estimated hazard ratio of existing and new customers was not significantly different from 1, so there was no significant difference between the two groups regarding time to the first OO.

For the zero-truncated negative binomial regression model, the results indicate that the COVID-19 incidence had a significantly positive effect on the number of OOs per cycle. The number among non-Hispanic black and other racial/ethnic groups was significantly lower than that among the non-Hispanic white group. No significant difference regarding the number of OOs per cycle was detected between customers with or without an infant, child, or woman participant. In addition, the number of WIC participants and the household size had no statistically significant effect on the number of repeat OOs. Existing consumers had a higher number of online orders per cycle than new consumers.

## 4. Discussion

This is one of the first quantitative studies to examine WIC OO behaviors among WIC households at a grocery chain level in the U.S. It contributes to the growing international literature on online grocery shopping, especially among low-income customers [19]. Studies in Jordan, Thailand, and China indicated that online grocery shopping has the potential to benefit customers with limited access to brick-and-mortar stores [20,21,22]. The perceived ease of use and social norms may promote the intention to adopt online grocery shopping. On the other hand, some factors may reduce the consumers’ interests in online grocery shopping. For example, a study in China indicated that potential concerns about the freshness of groceries purchased online may reduce their intention to shop for fresh produce online [23]. Moreover, there is still little research to examine the diffusion of online grocery ordering among low-income customers [24].

Since the WIC OO program is still in the pilot-testing stage, this study provides valuable insights regarding the behaviors of early WIC OO adopters in OK. A previous qualitative study reported that WIC customers had mixed feelings about WIC OO [25], which was confirmed in our quantitative study. Among these early adopters, 40.5% were one-timers, and the average time taken to place the first WIC OO was 57.1 days starting from 1 July 2020, when WIC OO was fully implemented at this OK WIC-approved vendor. Although some customers were identified as one-timers because the censured observation period ended in December 2020 and there was not enough time for them to place a second order, the majority of them could be due to a lack of interest in repeated OO in XYZ stores. A previous qualitative study also indicated that some WIC customers may have strong interests in OO and adopt it frequently [10], which was reflected in our finding that the OO accounted almost half of all XYZ visits in the study period. Therefore, this early experiment of WIC OO in XYZ shows that it has the value and potential to be replicated and adopted by other WIC-authorized vendors.

Although no significant disparities were detected regarding one-timer vs. repeat consumers across socio-demographics, non-Hispanic black WIC households were more likely to take a longer time to place their first OO, and the number of OOs was significantly lower than for non-Hispanic whites. More equalized access to WIC OO needs to be implemented and promoted so minority groups are not in a disadvantaged position in enjoying WIC technology innovations [26]. Moreover, existing XYZ customers were more likely to try out the new innovation at XYZ stores. Based on marketing theory, many technology innovations cannot reach the majority of the customers because of a diffusion “chasm” between the early adopters and mainstream customers [27]. It will be worth studying how to mobilize the existing customers as champions for new WIC technology innovations and to help the innovation reach across the diffusion chasm [26].

Since there is no guaranteed payment from the customer to the store when an OO is placed, the potential “no-show” or high rate of changes in orders can be a real threat to WIC vendors, who need to spend extra staff time to fulfill (or re-shelve) these orders. Although the preliminary results for this study confirmed some of the vendors’ concerns in this area, with an approximately 20% no-show rate, they alleviated other concerns due to a 77% no-item-change order rate. It will be important for future WIC OO experiments to identify the most effective ways to control no-show rates so that vendors will not waste staff time on such orders. Researchers can also conduct more thorough studies on the reasons for changed items, e.g., the unavailability of the ordered item or changed minds of WIC customers at pick-up. Further research also needs to be done on how to improve WIC customers’ redemption experiences while maintaining the proper business operational parameters in WIC vendors using the new WIC OO model [11].

Similar experiments in WIC OO were adopted in multiple states [11,18,28]. However, each vendor has developed its own WIC OO system, which can be a behavioral barrier for WIC customers. For example, if customers would like to visit multiple stores for WIC redemptions, they may need to adopt various WIC OO systems or apps to complete their orders. Since implementing WIC OO requires resources from the vendors, corner stores or small vendors may not have the resources and technology to adopt WIC OO technology, which puts them in a more disadvantaged position when competing against larger vendors. How to improve the WIC OO business model to facilitate WIC customers’ redemption remains a challenge to WIC agencies. The USDA WIC Taskforce on Supplemental Foods Delivery may lead the discussion among WIC vendors and agencies to identify any technology solutions. For example, integrating WIC OO into the existing WIC apps may serve as a gateway for WIC customers to conduct OO, which may eventually improve their WIC redemptions [29,30].

Since this is the first quantitative evaluation of WIC OO at the store chain level, the results need to be interpreted carefully, with due acknowledgment of their limitations. First, given the data limitation, this study is unable to identify other confounding factors that may affect customers’ redemption behaviors, e.g., individual customers’ COVID-19 status or the store environment. Second, the ordered or redeemed food is not always equal to the food consumed by the WIC participants. Third, the data do not include direct information on the nutritional value of the foods. Fourth, this time-sensitive study was conducted in a limited observation window during an ongoing pandemic. Fifth, since XYZ is a WIC-only store, the generalizability of the findings to other store types, such as supermarkets or convenience stores, may require future evaluation. Finally, XYZ customers were not intended to be representative of all WIC customers in OK or in the U.S.

Despite the above limitations, this study provides important evidence for policymakers, WIC-authorized vendors, and researchers who are eager to examine WIC OO behaviors in a pandemic. It builds a foundation for future research we hope to undertake to understand more about the reasons why WIC customers adopted or did not adopt the WIC OO option. Moreover, the food benefits ordered online need to be compared with those ordered in-store in order to determine whether OO is affecting food redemption choices and redemption rates. We are also working on another paper that focuses on the relationship between WIC OO adoption and the COVID-19 pandemic in OK.

Although the WIC program is unique among developed countries and the WIC OO technology is still in the pilot-test phase, this innovation can still provide a good example for developing countries who are eager to adopt app-based technology to promote nutrition assistance to mothers and children [31,32,33].

## 5. Conclusions

WIC OO is still in an early implementation stage and has been accepted by a small number of early adopters. Minority households took a longer time to adopt this innovation. Non-Hispanic black households had a fewer number of OOs compared with non-Hispanic white households. There were significant disparities in WIC OO adoption across socio-demographics.

## Figures and Tables

**Table 1:** Descriptive statistics of online ordering (OO) shoppers (OK-WIC-XYZ) (# of households = 726).

Variables	Number of Households	Repeated Consumers	One-Timer Consumers	*p ^1^*	# of Days Until the First Adoptions of WIC OO *^2^*	*p ^2^*	# of OO/Cycle *^2^*	*p ^2^*	Rate of OO (%) *^2^*	*p ^2^*
All	N = 726 *^1^*	n = 432 (59.5) *^1^*	n = 294 (40.5) *^1^*		57.1 (43.2)		2.8 (2.7)		46.9 (31.2)	
Racial/Ethnic group				0.31		0.09		0.15		<0.001
∙ Non-Hispanic White	172 (23.7)	110 (64.0)	62 (36.0)		61.6 (44.7)		3.3 (3.3)		61.2 (31.8)	
∙ Non-Hispanic Black	95 (13.1)	53 (55.8)	42 (44.2)		58.8 (44.5)		2.4 (1.9)		44.9 (29.7)	
∙ Hispanic	377 (51.9)	216 (57.3)	161 (42.7)		53.2 (41.5)		2.8 (2.6)		40.0 (28.7)	
∙ Others	82 (11.3)	53 (64.6)	29 (35.4)		63.9 (45.5)		2.6 (2.1)		51.5 (31.8)	
Having an infant participant?				0.69		0.45		0.66		0.73
∙ No	420 (57.9)	253 (60.2)	167 (39.8)		56.1 (43.0)		2.8 (2.6)		46.5 (30.5)	
∙ Yes	306 (42.1)	179 (58.5)	127 (41.5)		58.4 (43.6)		2.9 (2.8)		47.6 (32.0)	
Having a child participant?				0.06		0.11		0.03		0.02
∙ No	191 (26.3)	102 (53.4)	89 (46.6)		60.5 (43.4)		2.4 (1.9)		52.2 (33.4)	
∙ Yes	535 (73.7)	330 (61.7)	205 (38.3)		55.7 (43.1)		3.0 (2.9)		45.1 (30.1)	
Having a woman participant?				0.51		0.82		0.07		0.38
∙ No	475 (65.4)	278 (58.5)	197 (41.5)		57.1 (43.8)		2.6 (2.4)		46.0 (30.4)	
∙ Yes	251 (34.6)	154 (61.4)	97 (38.6)		57.1 (42.5)		3.2 (3.1)		48.7 (32.4)	
# of WIC participants				0.04		0.45		0.006		0.32
∙ 1	349 (48.1)	205 (58.7)	144 (41.3)		56.6 (42.9)		2.5 (2.1)		48.2 (30.4)	
∙ 2	261 (36.0)	146 (55.9)	115 (44.1)		60.2 (45.8)		2.9 (2.8)		46.5 (32.5)	
∙ 3 or more	116 (16.0)	81 (69.8)	35 (30.2)		52.3 (38.5)		3.7 (3.7)		44.0 (30.3)	
Household size				0.26		0.22		0.15		0.01
∙ 3 or less	310 (42.7)	183 (59.0)	127 (41.0)		59.7 (43.4)		2.7 (2.4)		50.7 (31.5)	
∙ 4 to 6	371 (51.1)	217 (58.5)	154 (41.5)		55.6 (43.2)		2.9 (2.9)		43.7 (30.5)	
∙ 7 or more	45 (6.2)	32 (71.1)	13 (28.9)		51.4 (42.0)		3.4 (2.8)		48.2 (31.8)	
Existing consumer				0.32		<0.001		0.18		<0.001
∙ No	151 (20.8)	84 (55.6)	67 (44.4)		79.1 (45.7)		2.4 (1.8)		68.2 (32.4)	
∙ Yes	575 (79.2)	348 (60.5)	227 (39.5)		51.3 (4.7)		2.9 (2.9)		41.3 (28.3)	

*^1^* Statistics presented: n (%); statistical tests performed: chi-square test of independence. *^2^* Statistics presented: mean (SD); statistical tests performed: Wilcoxon rank-sum test.

**Table 2:** Online orders characteristics across socio-demographics (# of orders = 2138).

Variables	Number of Households	Orders Exactly Match Picked Up (N = 1702)		Orders with Added Items but No Missing Items (*n* = 267)		Orders with Missing Items but No Added Items (*n* = 118)		Orders with Missing Items and Added Items (*n* = 51)	
		Avg Rate	*p*	Avg Rate	*p*	Avg Rate	*p*	Avg Rate	*p*
All	N = 2138	0.770 (0.339)		0.131 (0.266)		0.067 (0.208)		0.032 (0.148)	
Racial/Ethnic group			0.76		0.04		0.47		0.81
∙ Non-Hispanic White	597 (27.9)	0.758 (0.340)		0.158 (0.280)		0.045 (0.162)		0.039 (0.167)	
∙ Non-Hispanic Black	231 (10.8)	0.758 (0.361)		0.120 (0.283)		0.098 (0.260)		0.025 (0.129)	
∙ Hispanic	1086 (50.8)	0.776 (0.335)		0.118 (0.251)		0.072 (0.213)		0.034 (0.157)	
∙ Others	224 (10.5)	0.784 (0.335)		0.146 (0.282)		0.054 (0.200)		0.016 (0.066)	
Having an infant participant?			0.41		0.03		0.72		0.72
∙ No	1203 (56.3)	0.778 (0.335)		0.114 (0.248)		0.077 (0.224)		0.030 (0.143)	
∙ Yes	935 (43.7)	0.759 (0.346)		0.154 (0.287)		0.053 (0.182)		0.034 (0.155)	
Having a child participant?			0.30		0.004		0.003		0.55
∙ No	476 (22.3)	0.735 (0.369)		0.191 (0.327)		0.035 (0.154)		0.039 (0.165)	
∙ Yes	1662 (77.7)	0.783 (0.327)		0.110 (0.237)		0.078 (0.223)		0.029 (0.142)	
Having a woman participant?			0.08		0.001		0.30		0.39
∙ No	1298 (60.7)	0.785 (0.330)		0.108 (0.240)		0.077 (0.222)		0.030 (0.143)	
∙ Yes	840 (39.3)	0.742 (0.355)		0.174 (0.306)		0.048 (0.176)		0.037 (0.157)	
# of WIC participants			0.14		0.03		0.45		0.60
∙ 1	900 (42.1)	0.782 (0.339)		0.112 (0.250)		0.073 (0.219)		0.034 (0.155)	
∙ 2	787 (36.8)	0.740 (0.353)		0.161 (0.292)		0.064 (0.207)		0.035 (0.154)	
∙ 3 or more	451 (21.1)	0.802 (0.304)		0.121 (0.248)		0.057 (0.175)		0.020 (0.108)	
Household size			0.60		0.61		0.95		0.16
∙ 3 or less	871 (40.7)	0.785 (0.325)		0.122 (0.249)		0.067 (0.205)		0.027 (0.139)	
∙ 4 to 6	1106 (51.7)	0.758 (0.354)		0.137 (0.281)		0.067 (0.213)		0.038 (0.156)	
∙ 7 or more	161 (7.5)	0.765 (0.310)		0.143 (0.255)		0.069 (0.189)		0.022 (0.149)	
Existing consumer			0.69		0.07		0.02		0.17
∙ No	378 (17.7)	0.767 (0.354)		0.177 (0.312)		0.034 (0.150)		0.023 (0.128)	
∙ Yes	1760 (82.3)	0.771 (0.336)		0.119 (0.251)		0.076 (0.220)		0.035 (0.153)	

Statistics presented: n (%); mean (SD); statistical tests performed: Wilcoxon rank-sum test.

**Table 3:** Regression results of time to place the first online order and number of online orders.

Outcome	Time (days) Until First Placement of WIC Online Ordering			Number of Online Orders		
Model	Cox Proportional Hazards			Zero-Truncated Negative Binomial Regression		
Sample	OK-WIC-XYZ Households (*n* = 15,089)			Online Order Shoppers (*n* = 726)		
Variable	Hazard Ratio	95% CI *	*p*	B *	SE *	*p*
Racial/Ethnic group						
∙ Non-Hispanic White	Ref			Ref		
∙ Non-Hispanic Black	0.426	0.336, 0.540	<0.001	−0.374	0.140	0.007
∙ Hispanic	0.724	0.607, 0.863	<0.001	−0.172	0.099	0.08
∙ Others	0.570	0.443, 0.733	<0.001	−0.395	0.147	0.007
Having an infant participant? **						
∙ Yes	1.113	0.873, 1.419	0.39	−0.077	0.141	0.58
Having a child participant? **						
∙ Yes	1.371	1.021, 1.841	0.04	0.124	0.179	0.49
Having a woman participant? **						
∙ Yes	1.458	1.137, 1.869	0.003	−0.051	0.151	0.74
# of WIC participants						
∙ 1	Ref			Ref		
∙ 2	1.517	1.223, 1.882	<0.001	0.133	0.121	0.27
∙ 3 or more	1.872	1.231, 2.846	0.003	0.453	0.249	0.07
Household size						
∙ 3 or less	Ref			Ref		
∙ 4 to 6	0.761	0.654, 0.884	<0.001	−0.025	0.090	0.78
∙ 7 or more	0.621	0.454, 0.850	0.003	0.047	0.168	0.78
Existing consumer **						
∙ Yes	0.967	0.816, 1.146	0.70	0.322	0.109	0.003
log (County COVID-19)	0.210	0.190, 0.232	<0.001	1.168	0.069	<0.001
log (County population)	5.848	4.946, 6.915	<0.001	−1.181	0.086	<0.001

* CI: confidence interval; B: regression coefficient; SE: standard error. **: households with “No” as the reference group.

## Data Availability

The data presented in this study are not publicly available due to ownership restrictions.
